# *Opaque16*, a high lysine and tryptophan mutant, does not influence the key physico-biochemical characteristics in maize kernel

**DOI:** 10.1371/journal.pone.0190945

**Published:** 2018-01-08

**Authors:** Konsam Sarika, Firoz Hossain, Vignesh Muthusamy, Rajkumar U. Zunjare, Aanchal Baveja, Rajat Goswami, Nepolean Thirunavukkarasu, Sunil K. Jha, Hari S. Gupta

**Affiliations:** 1 Division of Genetics, ICAR-Indian Agricultural Research Institute, New Delhi, India; 2 Division of Post-harvest and Technology, ICAR-Indian Agricultural Research Institute, New Delhi, India; National Bureau of Plant Genetic Resources, INDIA

## Abstract

The enhancement of lysine and tryptophan in maize is so far basedon *opaque2*(*o2*) mutant, that along with the endosperm-modifiersled to development of Quality Protein Maize[QPM]. Though many mutants improving the endospermic protein quality were discovered, they could not be successfully deployed. Recently discovered *opaque16 (o16*)mutant enhances the lysine and tryptophan content in maize endosperm. In the present study, the influence of *o16* on the endosperm modification was analyzed in four F_2_ populations, two each segregating for *o16* allele alone and in combination with *o2*. The recessive *o16o16* seed endosperm was found to be vitreousphenotypically similar to wild-*O16O16*. The mutant did not influence the degree of kernel opaqueness in *o2o2* genetic background as opaqueness in *o2o2/O16O16* and *o2o2/o16o16* was similar. Grain hardness of *o16o16* was comparable with the normal and QPM maize. The pattern of microscopic organization of proteinaceous matrix and starch granules, and zein profiling of the storage protein in *o16o16* were found to be similar with normal maize endosperm, but distinct from the *o2o2*-soft genotype. The pattern in *o2o2/o16o16* was unique and different from *o2o2* and *o16o16* as well. Here we demonstrated the effects of *o16* on physico-biochemical characteristics of endosperm and report of *o16* possessing negligible influence on kernel modification and hardness, which holds a great significance in maize quality breeding programme.

## Introduction

Maize is one of the most important food cropsin sub-Saharan African, Latin American and many of the Asian countries[1].It is also an important source of poultry and livestock feed worldwide[2]. Storage protein of maize, prolamin also known as zein, constitutes about 70% of the total protein. Prolamin is characterized by limiting level of two essential amino acids, lysine and tryptophan[3,4]. Maize, therefore, being poor in nutritional quality does not provide balanced nutrition to human and mono-gastric animals such as poultry and pig. A mutation, *opaque2* (*o2*) discovered in 1920s was found to be nutritionally superior in lysine and tryptophan compared to normal maize [5]. However, the improvement in the quality was deterred by the pleiotropic effects of the mutant that causes soft endospermmaking the kernel more prone to insect infestation and pathogen susceptibility with poor processing quality and reduced yield[6]. Several other genetic mutations viz., *floury1* (*fl1*),*floury2*(*fl2*), *floury3*(*fl3*), *opaque5* (*o5*), *opaque6* (*o6*), *opaque7(o7*), *opaque15 (o15*), *Defective endosperm* (*Def-B30*), *Mucronate* (*Mc)* that affect the lysine content in maize endosperm, have been discovered[7]. Different combinations of these mutants to further increase the lysine and tryptophan were also tried, but could not succeed due to adverse pleiotropic effect that imposed severe constraints in implementing them[8, 9].

Researchers found that the opaqueness caused due to *o2* can be overcome with the accumulation of *o2*-modifiers and led to the development of Quality Protein Maize (QPM) with improved lysine content from 0.15 to 0.37% and tryptophan from 0.04 to 0.08% on average [10, 11]. The exact mechanism of the *o2* endosperm modification in QPM is not known but a possible role of 27-kDa γ-zein in recovering the vitreous phenotype has been put forward [12]. Genetic mapping of *o2* modifiers in QPM was found to be the locus encoding linked with 27-kDa γ-zein storage proteinson chromosome 7. Wu and Messing[13] later demonstrated that silencing of 27- and 16-kDa γ-zein genes resultin clumping of protein bodies and thus opacity of QPM seeds.

Yang et al.[14] discovered a recessive mutant from Robertson’s Mutator stocks and named it temporarily as *opaque16*(*o16*). The *o16* located on chromosome 8 induces higher lysine content compared to normal maize. The locus *o16* in *o2o2* genetic background increases lysine by ~30% over *o2o2* or *o16o16* alone. In our earlier studies, genotype with *o16o16* possessed nearly on average two-fold more lysine (0.247%) and tryptophan (0.072%) compared to normal maize (0.125% lysine and 0.035% tryptophan)[15]. The effect of *o16* on higher accumulation of lysine was also reported by Zhang et al.[16, 17]. Yang et al. [14] reported the presence of opaque phenotype in two *o16*-based inbreds. However, the effects of *o16* on degree of influence on endosperm opaqueness, hardness, zein profile and organization of starch granules with proteinaceous matrix in kernel in segregating populations have not been yet investigated. It is therefore, pertinent here to evaluate the performance of *o16* mutant on general endosperm attributes, as *o2* despite its nutritional superiority could not be initially accepted due to induction of soft endosperm. In the present study, we attempted to study the influence of *o16* on grain hardness and different physico-biochemical characteristics.

## Materials and methods

### Plant materials

The experimental materials consisted of four populations derived from two CIMMYT-based *o2o2* inbreds (CML161, CML193) and two CIMMYT-based normal (CML533 and CML537) inbreds crossed with an *o16o16*-donor line (QCL3024, a yellow line of Chinese origin). Derived F_1_s from the crosses were obtained from Guizhou Institute of Upland Food Crops, China. F_1_s of the four crosses were grown at the Indian Agricultural Research Institute, New Delhi, India during rainy season-2014. The F_2_ populations were raised at Winter Nursery Centre, Hyderabad of Indian Institute of Maize Research, New Delhi- during winter season 2014–15. Each of the F_2_ plants was selfed to generate F_3_ seeds. The derived F_3_ seeds along with three other inbreds: a CIMMYT-based normal inbred-CML543, a soft and opaque endosperm inbred-MGUQ-102 (*o2o2* based without endosperm modifiers), and a QPM inbred-HKI193-1(*o2o2* based with endosperm modifiers), were subjected for the studies.

### DNA isolation, PCR amplification and gel electrophoresis

Genomic DNA was extracted from young tender leaves by using CTAB method [18]. The PCR (Bio-Rad, California, USA) reaction was carried out applying ‘touch down’ procedure for 15 μl reaction mixture using REDtaq ReadyMix^TM^ PCR Reaction Mix (SIGMA-ALDRICH). 15 μl reaction mixture consists of 7.5 μl of REDtaqreaction mix, 3.5 μl water, 2 μl of DNA and 1 μl each of forward and reverse primers. The ‘touch down’ procedure consisted of three steps. The first step was set for 12 cycles: denaturation at 94°C for 30s, annealing at 62°C for 30s (reducing the annealing temperature subsequently by 0.5°C per cycle), and extension at 72°C for 45s. The second step was set for 45 cycles: denaturation at 94°C for 30s, annealing at 58°C for 45s, and extension at 72°C for 45s. The third stepfinal extension was carried out at 72°C for 7 min. The PCR amplicons of CML533-, CML537- and CML161-based populations were resolved in 4% agarose gel, while CML193-based population was resolved in 8% native PAGE acrylamide gel. The amplicon profiles were visualized in a gel documentation system (AlphaInnotech, California, USA).

### Genotyping

The genotyping of individual plant in each generation of all populations for *o2* was carried out using gene-based SSR markers, *phi112*, *phi057* and *umc1066*[19]and for *o16*,linked markers, *umc1141* and *umc1149*were used[14]. The test for hybridity of F_1_(s) and genotyping of individual plants in F_2_ generations were carried out by targeting these SSRs. Chi-square test was performed using MS-Excel 2010 for testing the goodness of fit between the segregation pattern at 5% level of significance.

### Endosperm modification

One hundred randomly selected seeds in each population were used for analyses of endosperm modification. The degree of opaqueness of seeds was analysed by using standard ‘light box’ with the formula: Degree of opaqueness = [(N_100_× 100) + (N_75_× 75) + (N_50_× 50) + (N_25_× 25) + (N_0_× 0)]/100, where N_100,_ N_75,_ N_50,_ N_25_ and N_0_ are the numbers of seeds with 100%, 75%, 50%, 25% and 0% opacity, respectively (Hossain et al. 2008). For observing the ratio of inner soft and outer hard endosperm, seed kernels were transversely cut through the centre by a sharp cutter exposing both the embryo and the surrounding tissue of endosperm.

### Grain hardness

Nine genotypic classes could be obtained in F_2_ derived F_3_ seeds of both crosses, CML161 × QCL3024 and CML193 × QCL3024 since the progenies are segregating for *o2* and *o16*. For the crosses, CML533 × QCL3024 and CML537 × QCL3024, where only *o16* was segregating, three classes could be obtained in F2 populations. Derived F_3_ families from F_2_ double homozygotes *viz*. *o2o2/o16o16*, *o2o2/O16O16*, *O2O2/o16o16*, and *O2O2/O16O16* were performed for grain hardness studies along with normal inbred CML543 (*O2O2/O16O16*), soft endosperm MGUQ-102 (*o2o2/O16O16*) and QPM lineHKI193-1 (*o2o2/O16O16*) as checks. Five randomly selected kernels per line were used for measuring grain hardness (GH) using Texture Analyzer (Scientific Microsystem, UK). The hardness was measured at grain moisture content of ~14%. A cylindrical probe of 75 mm diameter (P75 mm compression platen) was used. Individual seeds were placed centrally beneath the probe with the embryo facing down. The test speed of the probe was fixed at 2 mm/s and the compression distance at 70% with a trigger load cell of 500 kg. The first peak force (N, newton) in the force deformation curve was noted as GH of the seeds [20]. *t*-test was performed if the difference in hardness between the different classes and with the corresponding *O2O2*/*O16O16* in each populationis significant by using Microsoft Excel.

### Scanning electron microscopy of maize endosperm

Maize kernels were decapped and degermed with a razor blade and cut through the centre of the kernel giving a fracture with rough surface rather than a clean cut. A small piece from the central region of endosperm was used for study and was coated with an alloy of gold and palladium and documented in Zeiss EVO MA 10 Scanning electron microscope at 20kV/EHT and 80 Pa with a magnification of 1.50 KX.

### Protein profiling

The total protein and the zein fractions α-, β-, γ- and δ- zein fractions of different samples maize endosperm protein were extracted from 50 milligram of maize flour in accordance with Yue et al.[21]. The 10μlof extracted alcohol soluble zein protein fractions were profiled in 15% SDS-PAGE.

## Results

### Segregation of *o2* and *o16* through SSR markers analyses

**Fig 1:**
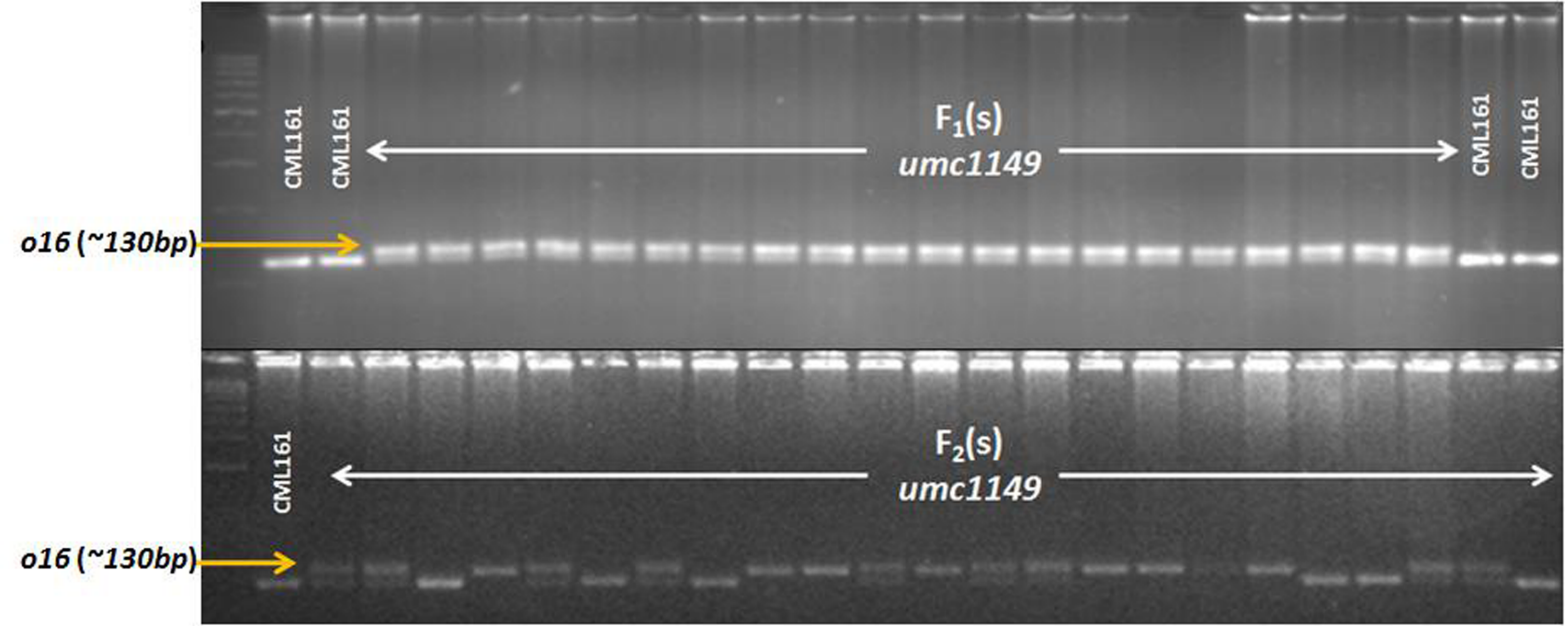
Marker segregation of *o16*-linked SSR *umc1149*. (A) F_1_s of the cross of CML161 × QCL3024 (B) F_2_ population derived from F_1_s of the cross of CML161 × QCL3024.

The three reported *o2*gene-based SSR markers viz., *phi112*,*phi057* and *umc1066* were used for testing the polymorphism between the female parents (CML161, CML193, CML533 and CML537) and the respective F_1_(s). Of the three, *umc1066* showed distinct polymorphismin 4% agarose gel, thus used for genotyping the F_2_ individual plants (Fig 1A). In the case of *o16*, Yang et al.[14] reported three linked SSRs viz. *umc1121*, *umc1141* and *umc1149*. InCML193 × QCL3024, *umc1141* showed a distinct polymorphism in 8% native PAGE and in the remaining three populations viz. CML161 × QCL3024, CML533 × QCL3024 and CML537 × QCL3024, *umc1149* was polymorphic in 4% agarose (Fig1B). The F_2_ populations of all the crosses exhibited a co-dominant segregation of both *o2* and *o16* as per Mendelian ratio of 1:2:1 (*p*< 0.05) (Table 1).

**Table 1:** Segregation pattern of SSRs associated with *opaque16* and *opaque2*.

	CML161 × QCL3024	CML193 × QCL3024	CML533 × QCL3024	CML537 × QCL3024
***opaque16***				
**Population size**	119	150	159	143
*o16o16*	30	39	41	40
*O16o16*	56	76	81	69
*O16O16*	33	35	37	34
**χ2**	0.563	0.3061	0.2579	0.6783
***p* value**	0.7546 ^ns^	0.8581 ^ns^	0.879 ^ns^	0.7124 ^ns^
***opaque2***				
***o2o2***	28	32	Na	na
***O2o2***	58	81	Na	na
***O2O2***	33	37	Na	na
**χ2**	0.4958	1.2933	Na	na
***p* value**	0.7804^ns^	0.5238 ^ns^	Na	na

ns: non-significant

Top row indicates the F_2_ populations derived from the respective crosses as mentioned; Genotyping was carried out by using *o2-*based marker *umc1066* and *o16*-linked marker *umc1149* in CML161 × QCL3024, CML533 × QCL3024, andCML537 × QCL3024 and *umc1141* in CML193 × QCL3024. ns- non significant; na- not applicable

### Effect of *o16* on the endosperm opaqueness

**Fig 2:**
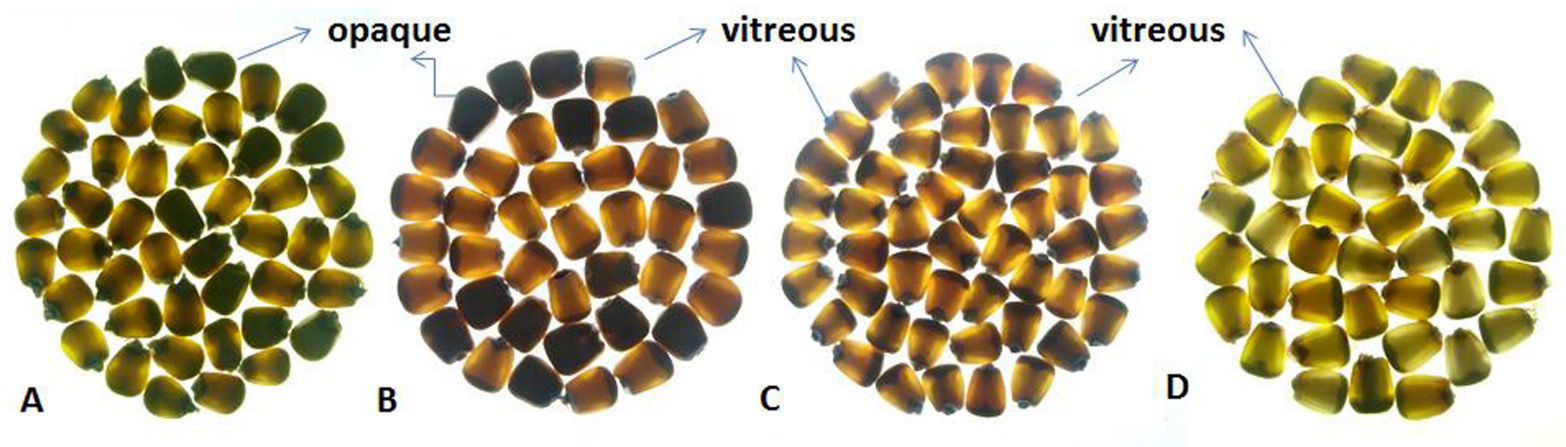
Light box testing of F_2_ seeds derived from crosses. (A) CML161 × QCL3024 (B) CML193 × QCL3024 (C) CML533 × QCL3024 and (D) CML537 × QCL3024.

**Fig 3:**
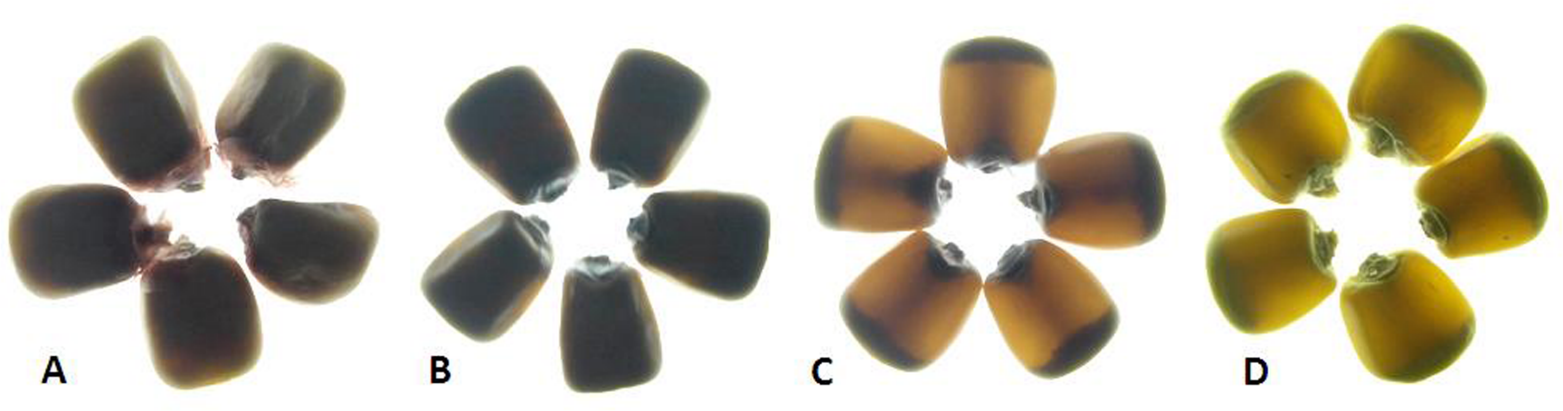
Light box testing of different F_3_ families seeds in the cross CML161 × QCL3024. (A) *o2o2*/*o16o16*(B)*o2o2*/*O16O16* (C) *O2O2*/*o16o16* (D) *O2O2*/*O16O16*.

**Fig 4:**
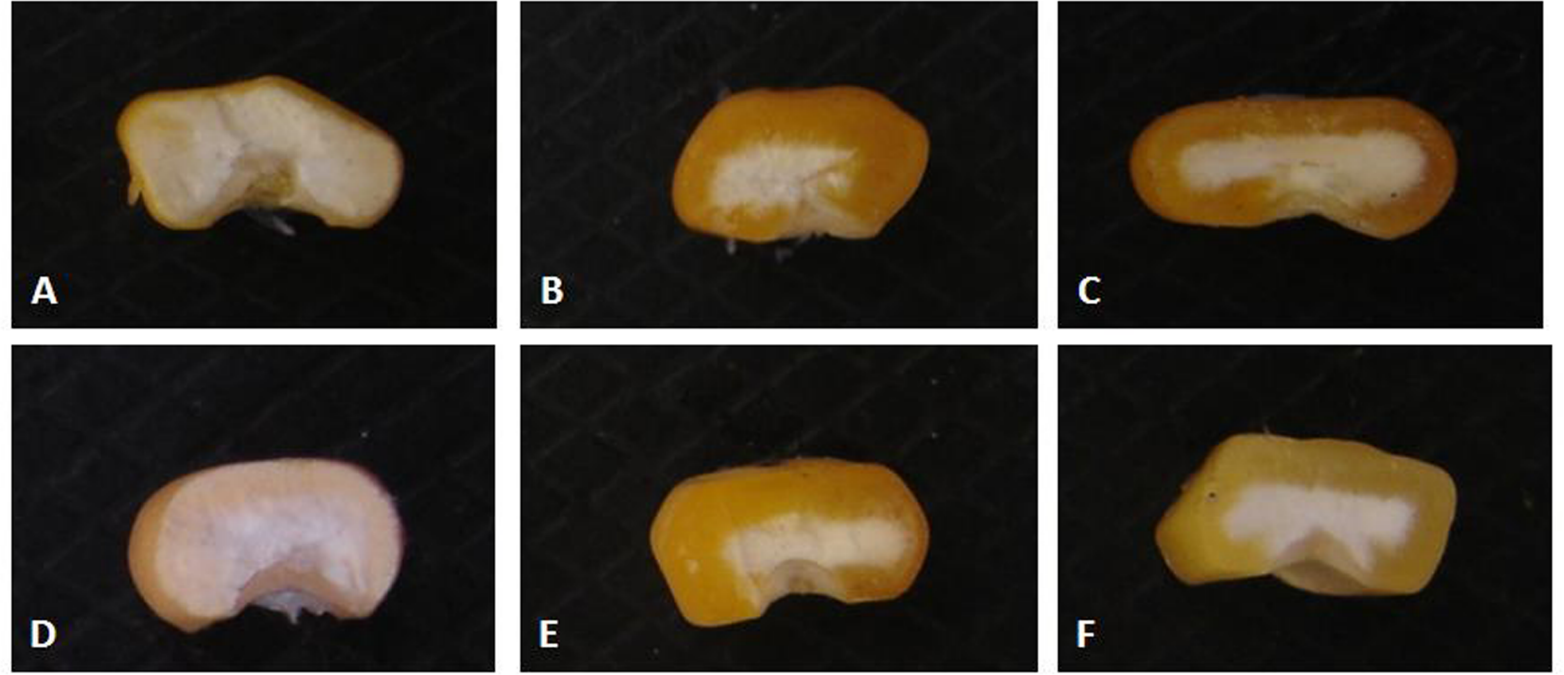
Ratio of hard and soft endosperm. (A) *o2o2*-soft and opaque line, MGUQ-102 (B)*O2O2* genotype normal line, CML543 (C)*o2o2*-modified QPM, HKI193-1 (D) *o2o2/o16o16* segregant (E-F) *O2O2/o16o16* segregants.

One hundredrandomly selected F_2_ seeds per cross were grouped into five classes with the scores in degree of opaqueness as 100%, 75%, 50%, 25% and 0%[22]. In CML161 × QCL3024 and CML193×QCL3024 (segregating for both *o2* and *o16*), the opaqueness in F_2_ generation was found to be 26.09% and 28.98%, respectively (Fig 2, Table 2). However, CML533 × QCL3024 and CML537 × QCL3024 segregating only for *o16* displayed a mere 2.25% and 0% opaqueness, respectively (Table 2). The extent of opaqueness in CML161 × QCL3024 and CML193 × QCL3024 F_2_-derived F_3_ seeds of genotype *o2o2/o16o16*(98.24% and 96.34%, respectively) was comparable to *o2o2/O16O16* (97.65% and 95.81%, respectively); genotype *O2O2/o16o16* (2.15% and 3.55%, respectively) and *O2O2/O16O16* (1.23% and 1.72%, respectively) displayed negligible opaqueness (Fig 3). In the case ofCML533 × QCL3024 and CML537 × QCL3024, the opaqueness observed in *o16o16*(4.30% and 0.35%, respectively) and *O16O16* (2.03% and 1.49%, respectively) was of similar degree (Table 3). The ratio of inner soft and outer hard endosperm of *o16o16* line was also found to be similar with the one observed in wild line CML543 and HKI193-1 QPM inbred (Fig 4).

**Table 2:** Average degree of opaqueness (%) in F_2_ seeds.

F_2_ populations	Parental genotypes	Opaqueness					
		0%	25%	50%	75%	100%	Average (%)
**CML161 × QCL3024**	*o2o2*/*O16O16*×*O2O2*/*o16o16*	67	4	7	0	22	26.09
**CML193 × QCL3024**		66	0	5	11	18	28.98
**CML533 × QCL3024**	*O2O2/O16O16*×*O2O2*/*o16o16*	91	5	2	0	0	2.25
**CML537 × QCL3024**		100	0	0	0	0	0

Hundred F_2_ seeds derived from selfed F_1_s of crosses mentioned in the left column were subjected to light box testing and scoring was done based on the degree of opacity

**Table 3:** Average degree of opaqueness (%) of F_3_ seeds.

Population	*o2o2*/*o16o16* (%)	*o2o2*/*O16O16*(%)	*O2O2*/*o16o16* (%)	*O2O2*/*O16O16* (%)
**CML161 × QCL3024**	98.24	97.65	2.15	1.23
**CML193 × QCL3024**	96.34	95.81	3.55	1.72
**CML533 × QCL3024**	NA	NA	4.30	2.03
**CML537 × QCL3024**	NA	NA	0.35	1.49

The F_3_ seeds derived from the F_2_ populations of crosses mentioned in the first column and their respective genotypes as mentioned in the top row were subjected for the light box testing and scoring was done based on the degree of opacity

### Effect of *o16* on grain hardness

The endosperm of genotypes *O2O2/o16o16* and *O2O2/O16O16* were hard, as reasonably force of higher degree was required to break the F_3_-grains of CML161 × QCL3024 (399.73N and 414.97N, respectively) and CML193 × QCL3024 (332.89N and 337.18N, respectively) compared to *o2o2/o16o16* and *o2o2/O16O16* (CML161 × QCL3024: 213.65N and 267.85N; CML193 × QCL3024: 205.52N and 246.96N), respectively (Table 4). Further, CML533 × QCL3024 and CML537 × QCL3024, segregating only for *o16*, showed a similar degree of hardness among families *O2O2/O16O16* and *O2O2/o16o16* and also with the normal line CML543 (*O2O2*) requiring 426.45N to break its grain. The same for HKI193-1 (QPM*-o2o2*) and MGUQ-102 (full opaque*-o2o2*) was 301.46 and 188.19N, respectively (Table 4).

**Table 4:** Force (N) required in breaking F_3_ seeds.

Populations	Genotypes	Newton (N)	*p*-value wrt to corresponding *O2O2/O16O16*
**CML161 × QCL3024**	*o2o2/o16o16*	213.65± 6.15	0.015^s^
	*o2o2/O16O16*	267.85 ± 5.18	0.002^s^
	*O2O2/o16o16*	399.73± 20.45	0.852^ns^
	*O2O2/O16O16*	414.97± 20.11	na
**CML193 × QCL3024**	*o2o2/o16o16*	205.52± 3.16	0.002^s^
	*o2o2/O16O16*	246.96± 12.45	0.005^s^
	*O2O2/o16o16*	332.89± 11.45	0.789^ns^
	*O2O2/O16O16*	337.18± 9.69	na
**CML533 × QCL3024**	*O2O2/O16O16*	312.25± 30.24	0.197^ns^
	*O2O2/o16o16*	378.34 ± 41.43	
**CML537 × QCL3024**	*O2O2/O16O16*	372.98 ± 30.59	0.787^ns^
	*O2O2/o16o16*	423.12± 32.14	
**CML543 (Normal)**	*O2O2/O16O16*	426.45 ± 21.56	na
**MGUQ-102 (Full opaque)**	*o2o2/O16O16*	188.19 ± 13.33	na
**HKI193-1(QPM)**	*o2o2/O16O16*	301.06 ± 19.04	na
*SE*		21.18	na

s: significant; ns: non-significant

Grain hardness analyses of F_3_ seeds derived from the F_2_ plants genotyped as mentioned in the middle column were carried out with the Texture Analyser. The force (N) required to break each grain were recorded. The last column indicates the mean force required to break seeds of the respective genotypes for each crosses

### Effect of o16 on organization of starch granules and proteinaceous matrix

**Fig 5:**
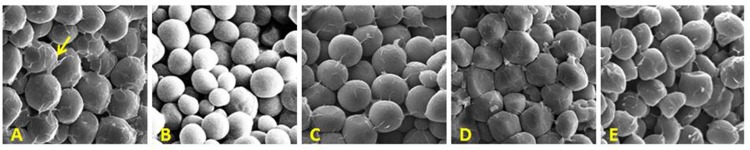
Microscopic view of protein bodies and starch granules arrangement under SEM. (A) *O2O2* genotype normal line, CML543 (B) *o2o2*-soft and opaque line, MGUQ-102 (C) *o2o2*-modified QPM, HKI193-1 (D) *o16o16* genotype (*opaque16* line) (E) *o2o2/o16o16* genotype (double mutant) (Yellow arrow: proteinaceous matrix spreading over the round starch granules).

The morphological arrangement of the starch granules and proteinaceous matrix were compared among *O2O2* (CML543), *o2o2*(MGUQ-102), *o2o2*-modified (HKI193-1), and *o16o16* and *o2o2/o16o16*F_3_ seeds. It revealed that the starch granules of normal line had an angular polygonal shape with proteinaceous matrix surrounding them, and characterized by a tightly packed structure with no air space (Fig 5A). But a significant reduction in the proteinaceous matrixadhering to the starch granules was observedin the soft endosperm line, MGUQ-102(Fig 5B); the starch granules wereloosely packed with relatively large intergranular space between starch granules. In HKI193-1, though the starch granules were spherical and smooth, a relatively more proteinaceous matrix adhered to the starch granules with lesser air space revealing a tighter interaction among the starch granules of seed endosperm (Fig 5C). The *o16o16* line had more or less similar microscopic arrangement with that of a normal line with angular polygonal shape starch granules and air tight packed structure with proteinaceous matrix (Fig 5D). The structure of starch granules of the genotype *o2o2/o16o16* (Fig 5E) was intermediate between *o2o2* (Fig 5B) and *o16o16* (Fig 5D), having semi-polygonal shape with spare proteinaceous matrix and less packed compared to *o16o16*.

### Effect of o16 on zein protein fractions

**Fig 6:**
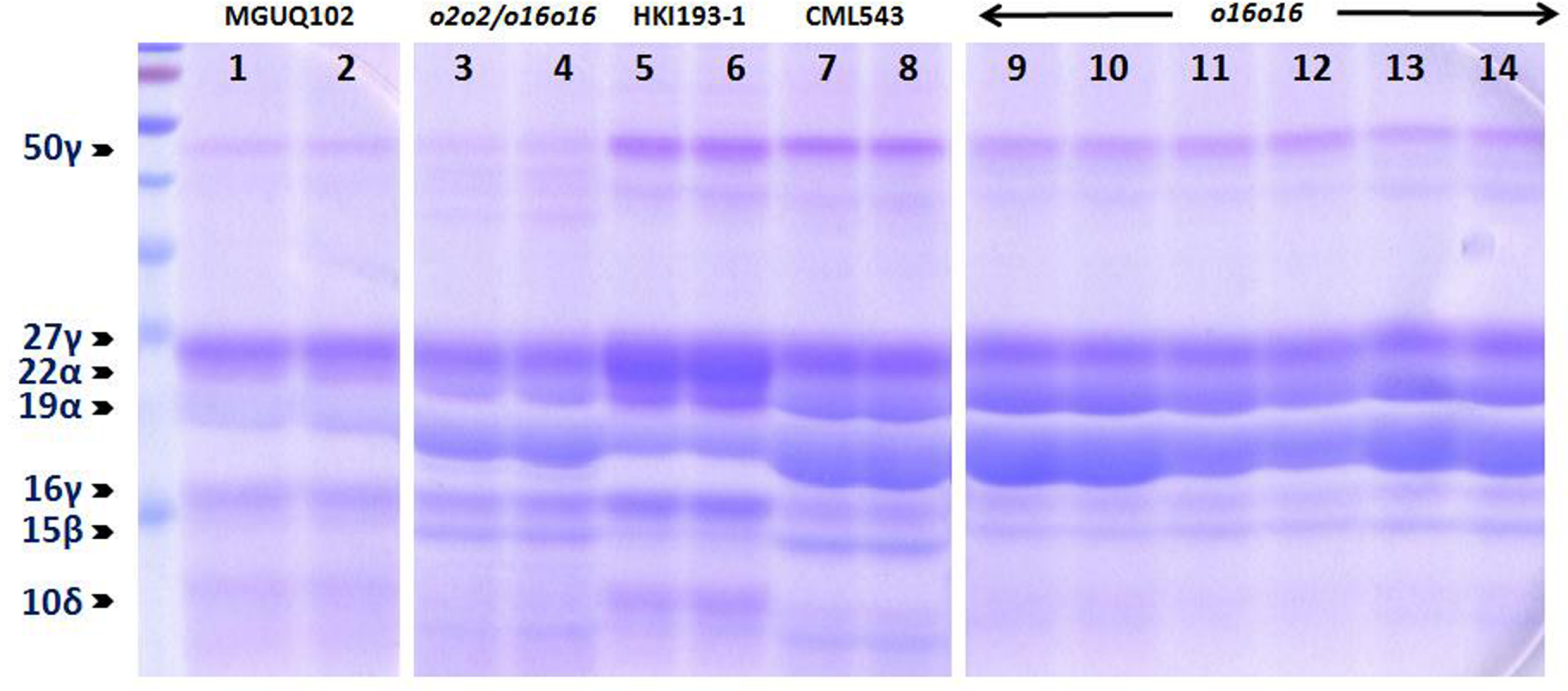
SDS-PAGE analysis of components of zein proteins in *o2o2*-soft and opaque line, MGUQ-102 (1 & 2 lane); *o2o2/o16o16* (3 & 4 lane); *o2o2*-modified QPM, HKI193-1 (5 & 6 lane); *O2O2* genotype normal line, CML543 (7 & 8 lane) and different *o16o16* lines (9–14 lane). The profiling had been done with two replications for each genotype.

The variation in zein protein profile among *o2*, *o16* and wild type genotypes could be observed in Fig 6. The fully opaque-*o2o2* (MGUQ-102) showed a considerable reduction in both 19- and 22-kDa α-zein. We could also observe a nearly two-fold increase in the expression of 16-, 27- and 50-kDa γ-zein in modified-*o2o2* (QPM: HKI193-1) compared to fully opaque *o2o2-*soft line, MGUQ-102. The *o16o16* genotypes showed a very similar profile with that of the normal line, CML543 but with a slight reduction of 50-kDa γ-zein and 15-kDa β-zein. However, it showed a completely different pattern from MGUQ-102 with a higher level of expression in 19- and 22-kDa α-zein, but a similar expression of 27-kDa γ-zein. The zein profile of *o2o2/o16o16* was unique with intermediate levels of 19- and 22-kDa α-zein as compared to *o2o2*-soft and *o16o16*. However, it possessed less 50-kDa γ-zein compared to *o2o2*-soft, and more levels of 15-kDa β-zein as found in *o16o16*. The 16- and 27-kDa γ-zein were similar to both *o2o2*-soft and *o16o16* type.

## Discussion

Recessive *o2* gene-based SSR *umc1066* confirmed the true hybridity of F_1_s with a perfect Mendelian segregation of 1:2:1 in F_2_ populations (*p*<0.05). It has been relied upon for genotyping individual plant positive for *o2* allele in earlier studies of several breeding programme [11, 14]. *o16* linked-SSR, *umc1149* showed perfect segregation in CML161 × QCL3024, CML533 × QCL3024 and CML537 × QCL3024 but failed to do so in CML193 × QCL3024. However, *umc1141* showed a distinct polymorphism inCML193 × QCL3024 in 8% native PAGE and were therefore used for further genotyping. Yang et al. [23] and Zhang et al. [17] used *umc1141* for selecting the individuals possessing *o16* allele. The *o2* based *umc1066*and *o16* based *umc1141* and *umc1149*SSR markers were successfully used in genotyping the present study’s F_2_ populations, and in classifying the individual plants into different genotypic classes for further physico-biochemical studies.

Phenotypic screening of individual seed opacity under light box is the most convenient and efficient strategy for studying the endosperm modification. The significant degree of opaqueness in F_2_ seeds of populations where both *o2* and *o16* were segregating and the non-significant in populations, where *o16* was segregating alone suggested that *o16* did not influence endosperm modification significantly as opposed to *o2* which induces various degree of endosperm opaqueness. The average opacity in the two *o2* and *o16* segregating F_2_ populations (26.09% and 28.98%) is expected if *o2* alone is affecting the modification and segregating in the ratio of 3 vitreous/translucent: 1 opaque [24](Table 2). This was further confirmed through F_3_ seed analyses where the F_2_-derived *o16o16* showed a negligible opacity and F_2_-derived *o2o2/o16o16* showed full opacity of endosperm. Therefore, *o16* alone possesses negligible effects (0.35–4.30% opaqueness) on inducing opaqueness. In contrast, Yang et al. [25] reported *o16*-based inbreds *viz*., QCL3024 and QCL3021 having opaque phenotype in endosperm, however, the extent of opaqueness has not been mentioned. Grain hardness corresponds the kernel density and determines the resistivity towards storage pests infestation and fungal infection [26, 27].Similar hardness observed in *O2O2/o16o16* and *O2O2/O16O16* genotypes derived F_3_ seeds with wild type inbred (CML543) and more hardness than the *o2o2*(MGUQ-102) and *o2o2/o16o16* segregants as well clearly demonstrated that *o16* alone did not induce softness in the endosperm. However, the degree of softness in *o2* genetic background is determined by the presence of modifier loci. In the case of *o2o2/o16o16* and *o2o2/O16O16*, grains were almost entirely soft; much favourable modifiers may be absent in the genetic background. However, grains of QPM were much harder due to the presence of favourable modifier loci [8, 22]. The *o16* therefore, did not have any negative impact on the endosperm hardness unlike *o2* which generally inflicts softness in the kernel. This was also evident from the proportion of hard- (orange or yellow translucent portion) and soft- (white portion) endosperm in the grains of *o2o2*-soft, QPM, normal (*O2O2*) and *o16o16* genotypes (Fig 4).

During desiccation of seeds, rough endoplasmic reticulum membranes break down exposing the zeins protein mixing with the other content of the cytoplasm. It acts as cementing glue thereby providing an airtight interaction with starch granules in normal vitreous seed endosperm in wild maize endosperm [13, 28]. Angular polygonal shape starch granules with surrounding proteinaceous matrix making them a tightly packed structure with no air space, similar to the normal maize endosperm, *o16o16* exhibited a vitreous texture of endosperm. This also explained the similarity observed in the grain hardness of *o16o16* genotypes with normal line, CML543. The compact protein bodies and its interaction with starch granules through amorphous, non-crystalline amylopectin molecules at the surface links starch granules together, and makes the packaging more compact and grain appearance as vitreous [12, 28]. In the case of soft and opaque endosperm line, MGUQ-102, the protein matrix was scanty owing to weak interaction with the starch granules, followed by the large intergranular space making the endosperm loosely packed. The opacity is due to the diffraction of light caused by the air spaces left due to loose packaging of protein and starch granules in the endosperm [13]. QPM seeds showed more vitreous and hard due to accumulation of *o2* modifiers in the genetic background (Table 4) [28] and with more of proteinaceous matrix as compared to MGUQ-102. The compact packaging of starch and protein bodies in *o16o16* thus conferred vitreous kernels, while the air space left due to weak interaction made the kernels of *o2o2* and *o2o2/o16o16* as soft and opaque.

SDS-PAGE was used to compare qualitatively and to some extent quantitatively as well for prolamin fraction in the lines [29]. Similar profile of *o16o16* genotypes with the normal line further strengthens the finding of *o16* having similar grain hardness and vitreous grain endosperm with the wild normal maize line, CML543. However, it showed a completely different pattern from *o2o2-*soft line with higher level of expression in 19- and 22-kDa α-zein, but similar expression of 27-kDa γ-zein. The zein profile of *o2o2/o16o16* was unique with intermediate levels of 19- and 22-kDa α-zein as compared to *o2o2*-soft and *o16o16*. Considerable reduction in both 19- and 22- kDa α-zein in *o2o2* individual had been observed in earlier studies [30]. Two-fold increase in the expression of 16-, 27- and 50-kDa γ-zein in modified-*o2o2* has been identified as the major factor in endosperm modification [28]. Several studies demonstrated a positive relationship between the content of 27-kDa γ- zein and endosperm vitreousness [31]. Segal et al. [32] induced a full opaque kernel phenotype by silencing the 22-kDa α- zeins by RNAi, while the overproduction of 27-kDa γ-zein enhanced protein body number resulting with more vitreous phenotype in QPM [33]. The disulfide bonds of cystein residues in γ-zein helps in extensive cross-linking and covalent linkage between protein bodies could provide a mechanism for cementing protein bodies around starch grains [34].

The findings here thus establish that the mechanism of higher synthesis of lysine and tryptophan in *o16* mutant is entirely different from the *o2*. The higher accumulation of lysine and tryptophan might be due to regulation of genes operating in amino acid biosynthesis pathway, or other unknown mechanisms. *O2* located on chromosome 7 codes for a DNA binding protein belonging to basic leucine zipper class of transcriptional factors, and acts as transcriptional activator of 19- and 22-kDa α-zein genes [35, 36]. The mutant *o2-*based proteininduces an overall reduction of 50–70% in zein protein which increases non-zein proteins proportionally, resulting inan increase of lysine content twice than that in normal maize [37]. The mechanism behind the enhanced nutritional value of o16 needs further investigation since zein profile of *o16o16* differs considerably from *o2o2*. It is worth mentioning that among the various discovered high lysine mutants, only *o2*, *fl2* and *Def-B30* affect different aspects of storage protein synthesis and alter zein content and compositions [38]. The other mutants such as *o5*, *o15*, *fl1*, *Mc* do not induce significant changes in zein content and composition suggesting that additional factors are also important in determining the kernel texture[39]. The *o15* mutation exerts its effect primarily on the 27-kDa γ-zeins [40]. The *fl1* mutation is rather resulted due to abnormal placement of α-zeins within the protein bodies. *Fl1* encodes a transmembrane protein that is located in the protein body ER membrane [41]. Similarly, *o5* mutant phenotype is caused by a reduction in the galactolipid content of the maize endosperm, with no change in zein proteins [42].

The novel high lysine and tryptophan mutant *o16* thus possessed no adverse effect on the endosperm modification. The recessive *o16* alone improves the nutritional quality of maize and can be utilized as effectively as *o2*[15]. Thus, it holds a significant promise in quality breeding programme. QPM breeding programme has traditionally used *o2* coupled with modifier for enhancement of lysine and tryptophan. However, the challenge remains in accumulation of favourable modifiers in *o2* genetic background to impart kernel hardness [8, 22]. Since the *o16o16* genotypes possessed vitreous endosperm and equivalent grain hardness to normal line, the mutant provides a tremendous advantage to the breeders as accumulation of modifiers in the genetic background need not be looked into while breeding for high lysine and tryptophan. The pyramided genotype *o2o2/o16o16* has higher lysine and tryptophan over *o2o2* alone [14]. So in this case of double mutant combination, accumulation of modifier loci would remain the challenge during the line development. However, several QTLs for these modifiers have recently been identified and diverse set of QPM inbreds have been characterized using SSRs linked those loci11. Availability of SSRs associated with *o2*, *o16* and QTLs linked to modifier loci provide great opportunity to undertaken marker-assisted selection to develop high lysine and tryptophan maize with hard endosperm; it can be further used to fine map the *o16* locus, and through chromosome walking the sequence of *o16* can be derived. Besides, gene silencing approach may also lead to the cloning and characterization of the *o16* locus. Though in the present study, *o16* was not characterized at sequence and transcript/polypeptide level, the information generated here on its effect on kernel attributes are of paramount importance in QPM breeding programme. This is first ever study reported on the effect of *o16* on kernel hardness, zein protein profiles and microscopic arrangement of starch granules with proteinaceous matrix.
